# Dynamic regulation of pancreatic β cell function and gene expression by the SND1 coregulator *in vitro*

**DOI:** 10.1080/19382014.2023.2267725

**Published:** 2023-10-15

**Authors:** Sukrati Kanojia, Rebecca K. Davidson, Jason M. Conley, Jerry Xu, Meredith Osmulski, Emily K. Sims, Hongxia Ren, Jason M. Spaeth

**Affiliations:** a Department of Biochemistry & Molecular Biology, Indiana University School of Medicine, Indianapolis, IN, USA; b Center for Diabetes & Metabolic Diseases, Indiana University School of Medicine, Indianapolis, IN, USA; c Herman B. Wells Center for Pediatric Research, Indiana University School of Medicine, Indianapolis, IN, USA; d Department of Pediatrics, Indiana University School of Medicine, Indianapolis, IN, USA

**Keywords:** Coregulator, insulin secretion, SND1, transcription, transcription factor, type 2 diabetes, β cell

## Abstract

The pancreatic β cell synthesizes, packages, and secretes insulin in response to glucose-stimulation to maintain blood glucose homeostasis. Under diabetic conditions, a subset of β cells fail and lose expression of key transcription factors (TFs) required for insulin secretion. Among these TFs is Pancreatic and duodenal homeobox 1 (PDX1), which recruits a unique subset of transcriptional coregulators to modulate its activity. Here we describe a novel interacting partner of PDX1, the Staphylococcal Nuclease and Tudor domain-containing protein (SND1), which has been shown to facilitate protein-protein interactions and transcriptional control through diverse mechanisms in a variety of tissues. PDX1:SND1 interactions were confirmed in rodent β cell lines, mouse islets, and human islets. Utilizing CRISPR-Cas9 gene editing technology, we deleted *Snd1* from the mouse β cell lines, which revealed numerous differentially expressed genes linked to insulin secretion and cell proliferation, including limited expression of *Glp1r*. We observed *Snd1* deficient β cell lines had reduced cell expansion rates, GLP1R protein levels, and limited cAMP accumulation under stimulatory conditions, and further show that acute ablation of *Snd1* impaired insulin secretion in rodent and human β cell lines. Lastly, we discovered that PDX1:SND1 interactions were profoundly reduced in human β cells from donors with type 2 diabetes (T2D). These observations suggest the PDX1:SND1 complex formation is critical for controlling a subset of genes important for β cell function and is targeted in diabetes pathogenesis.

## GRAPHICAL ABSTRACT

**Figure:**
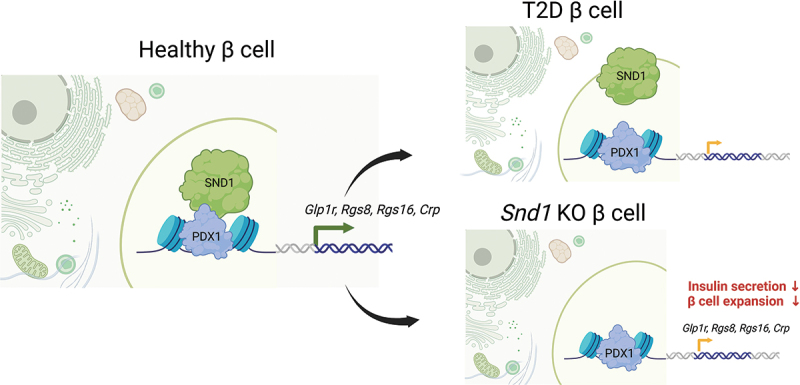

## Introduction

Transcriptional coregulators constitute a pivotal component of gene regulation within eukaryotic systems. Their precise recruitment to DNA-bound transcription factors (TFs) elicits either positive or negative impacts on gene transcription. Varied classes of coregulators operate to: 1. Modify chromatin structure, thereby rendering it accessible or restrictive to other proteins through both enzymatic and non-enzymatic mechanisms^1^, 2. Forge physical connections between DNA-bound TFs and the broader transcriptional machinery via protein-protein interactions^2^, or 3. Covalently alter histone tails through processes such as acetylation, methylation, glycosylation, phosphorylation, ubiquitination, and sumoylation^3^. Each of these functions is executed in a gene-specific manner, entailing interactions with DNA-bound TFs that hinge on cellular cues for their recruitment and activity.

Pdx1 is one of the most influential TFs in the pancreas, as it plays a central role in pancreatogenesis, islet β cell development and function and maintenance of β cell identity^4–6^. For example, *PDX1* is one of the diabetes-associated genes in which homozygous mutant humans exhibit pancreatic agenesis^7^, while heterozygotes develop early-onset diabetes consistent with both a developmental and functional role for PDX1^8–11^. Moreover, conditional removal of Pdx1 from mature mouse β cells results in severe glucose dyshomeostasis that stems from rapid deterioration of gene expression programs essential for maintaining β cell function and identity^4^. Inactivation of islet-enriched TFs is associated with and Type 2 diabetes (T2D) pathogenesis. For example, gene expression and immunofluorescence analyses revealed that expression of PDX1, NKX6.1 and MAFA, a sensitive PDX1 gene target are reduced in islets from human donors with T1D and long standing T2D^12^. Given the influential role of Pdx1 in islet ontogeny and β cell function, as well as the control Pdx1 has on the transcriptional properties to maintain β cell identity, understanding ways in which Pdx1 transcriptional activity is modulated is paramount to define its role in diabetes pathogenesis.

Previous work from our group and others has demonstrated the importance of PDX1-associated coregulators in modulation of its transcriptional activity in pancreas development and the mature β cell. An unbiased mass spectrometry screen *in vitro* revealed Pdx1 interacts with a diverse group of chromatin modifiers, including the Ruvbl1/2 DNA helicase, ATP-dependent SWI/SNF chromatin remodeling complex, the chromodomain helicase DNA-binding protein 4 (CHD4) subunit of the Nucleosome Remodeling and Deacetylase (NuRD) complex^13^, and the Staphylococcal Nuclease And Tudor Domain Containing 1 (SND1) coregulator (Personal Communication, Roland Stein, Vanderbilt University (April 2022)). The SWI/SNF chromatin remodeling complex was shown to control pancreatic progenitor cell proliferation and regulate insulin production by permitting PDX1 association with the *Ins2* enhancer^14^. Furthermore, the CHD4 subunit of the NuRD complex was found to regulate glucose homeostasis *in vivo* and *MafA* expression by controlling *MafA* Region 3 enhancer accessibility^15^. Here our focus is on interrogating the role of the SND1 coregulator on controlling gene expression and β cell function *in vitro*.

SND1 contains four repeated staphylococcal nuclease domains and a single Tudor domain, which comprises its endonuclease activity and ability to interact with individual proteins, coregulatory complexes and nucleic acids^16,17^. SND1 has been shown to modulate the transcriptional activity of the STAT5 and STAT6 TFs by controlling inflammation induced gene expression responses in B-lymphocytes^18,19^. In preadipocytes, PPARγ activity is regulated by Snd1 to promote adipogenesis^20^. In these settings, Snd1 acts as a coactivator where it bridges TFs with components of the RNA polymerase II machinery. SND1 has also been shown to govern chromatin accessibility in ovarian cancer cell lines through recruitment of histone acetyltransferases p300 and GCN5^21^. Additionally, SND1 has garnered attention as a metastatic cancer biomarker given its positive correlation with cancer progression^22^. Herein, we characterize the role of Snd1 in controlling β cell function. We demonstrate that PDX1 and SND1 interact in rodent and human β cells, where reduced levels of Snd1 negatively influence insulin secretion. β cell expansion and gene targets critical for β cell function are markedly affected in cells deficient for SND1. Lastly, we reveal interactions between PDX1 and SND1 are reduced in T2D donor β cells and discuss the implications of our findings in relation to T2D pathogenesis.

## Materials and methods

### Cell culture

Rodent and human β cell lines: mouse βTC3 cells, mouse insulinoma 6 (MIN6) cells, rat INS-1 832/13 and human EndoC-βH1 cells (passage number 65–70 used) were cultured using protocols as previously described^23^.

### Snd1 knockout cell lines preparation

Cas9-expressing MIN6 cells were generated as previously described^23^. To generate lentivirus expressing *Snd1* specific gRNAs, we transfected HEK2 93T cells with packaging plasmids pVSV-G, psPAX2 and puromycin-resistant single guide gRNA transfer vectors (Addgene clones: 138479, 12260 and 52,963 (used as backbone for cloning gRNAs)) (Table 1) in Opti-MEM using Lipofectamine 2000. Lentivirus containing *Snd1* gRNA was used to infect MIN6-Cas9 cells which were subsequently selected with Puromycin. One week later, surviving cells were seeded at single cell density, expanded, and screened for SND1 by western blot analyses. We generated 3 *Snd1* knockout clones along with 3 *intron* targeted control cell lines. Sanger Sequencing of Snd1 knockout cell lines was performed to confirm indel of *exon 2*.

**Table 1.** gRNA sequence information.

lentiGuide-Puro sgRNA	
gRNA Control *Snd1* Intron	TTTTGGCTCAACCAACGCAAAGG
gRNA1 *Snd1* Exon 2	GGTGCGCCATAATTGTCCGAGGG
gRNA 2 *Snd1* Exon 2	AATTGTCCGAGGGCAGCCCCGGG

### RNAi-mediated knockdown in β cell lines

Knockdown of *Snd1* was performed as previously described^23^ using the following small interfering (si)RNA for rodent (INS-1 832/13) and human (EndoC-βH1): ON-TARGETplus mouse *Snd1* (Dharmacon, Catalog ID: L-049812-01-0005), human *SND1* (Dharmacon, Catalog ID: L-010657-01-0005), nontargeting control (Dharmacon, Catalog ID:D-001810-10-05). After transfection, RNA and nuclear extract were collected at 48 hours for INS-1 832/13 cells and at 72 hours for EndoC-βH1 cells. The nuclear extracts were fractionated following the procedure previously described^23^. PVDF membranes were then probed with rabbit α-SND1 antibody (Invitrogen, PA5–40124; 1:500) and mouse α-β-actin antibody (Santa Cruz, sc -47,778; 1:1000). The LI-COR system was utilized in conjunction with secondary antibodies to capture images of the blots. The experiments were repeated at least three times, and ImageJ software was employed to quantitate the results.

### Animals and tissue preparation

All animal studies were reviewed and approved by the Indiana University Institutional Animal Care and Use Committee. Mice were housed and cared for according to the Indiana University Laboratory Animal Resource Center and the Institutional Animal Care and Use Committee/Office of Animal Welfare Assurance standards and guidelines. Twelve-week-old male C57BL/6J mice (Jackson Laboratories) were used to isolate whole mouse pancreata. The pancreata were fixed in 4% (v/v) paraformaldehyde/PBS for 4 hours, washed, and then embedded in O.C.T. embedding medium. Cryosections of 6 μm thickness were obtained using a Leica Cryostat. Human pancreatic tissue sections were obtained from the Network for Pancreatic Organ Donors with Diabetes (nPOD) (Table 2).

**Table 2.** nPOD donor sample information.

Case #:	RRID	Diabetes Status	Age (years)	Sex	Race	BMI (kg/m^2^)	T2D Duration (years)	Clinical Information
6020	SAMN15879077	No diabetes	60	Male	Caucasian	29.8	-	intracerebral hemorrhage
6095	SAMN15879152	No diabetes	40	Male	Hispanic/Latino	35.5	-	traffic accident
6254	SAMN15879310	No diabetes	38	Male	Caucasian	30.5	-	anoxic brain injury
6168	SAMN15879224	No diabetes	51	Male	Hispanic/Latino	25.2	-	acute ischemic stroke
6297	SAMN15879351	T2D	60	Male	Caucasian	29.5	3	pulmonary embolism
6203	SAMN15879259	T2D	68.6	Male	Caucasian	32.5	5	intracerebral/intraventricular hemorrhage
6255	SAMN15879311	T2D	55	Male	Caucasian	29.35	6	intraparenchymal hemorrhage
6124	SAMN15879181	T2D	62.3	Male	Caucasian	33.7	3	stroke

### Co-immunoprecipitation and western blotting

To conduct co-immunoprecipitation experiments, 100 μg of either βTC3 or INS-1 832/13 nuclear extracts was combined with goat α-PDX1 antibody (Abcam, ab47383), α-SND1 antibody (PA5–40124), and either goat or rabbit IgG. The mixture was then incubated overnight at 4°C. The following day, Protein A/G PLUS-agarose was used to precipitate the antibody complexes, which were subsequently washed with PBS and boiled with SDS loading dye. The immunoprecipitants were separated by SDS-PAGE, transferred to PVDF membranes, and blocked with Blocking Buffer (LI-COR, 927–70001). Membranes were probed with primary antibodies: goat α-Pdx1 (Abcam, ab47383; 1:20000) and rabbit α-SND1 (Abcam, ab65078; 1:500). For detection, LI-COR IRDye secondary antibodies were employed, and fluorometric scanning was carried out using an Odyssey CLx Imager. Finally, ImageJ software was utilized to quantify the band intensity.

### Proximity ligation assay and immunofluorescence

EndoC-βH1 cells were fixed with 4% paraformaldehyde and permeabilized with 0.1% Triton X-100 24 hours after seeding, before blocking and adding primary antibodies. For mouse cryosections, tissue sections were washed and subjected to 1% SDS-mediated permeabilization prior to blocking and adding primary antibodies. For paraffin-embedded human pancreatic sections from nPOD donors, heat-activated antigen retrieval (Vector Laboratories, H-3300) was performed before blocking and adding primary antibodies.

The proximity ligation assays (PLAs) were conducted following the guidelines provided by the manufacturer (Sigma-Aldrich, DUO92105). The primary antibodies used for proximity ligation were goat α-Pdx1 (Abcam, ab47383; 1:10000) and rabbit α-SND1 (Abcam, ab65078; 1:500). Guinea pig α-insulin (Dako, A0564; 1:100) and mouse α-proinsulin targeting human proinsulin (DSHB, GS-9A8s; 1:200) were utilized for tissue sections. The detection of insulin and human proinsulin was performed using Cy2 secondary fluorophores. Immunofluorescence Z-Stack images were acquired through the depth of the tissues/cells using a Zeiss LSM 800 confocal laser scanning scope and processed using ImageJ software. To quantify PLA signals in human tissue sections, we manually enumerated the signals from individual Z-stack images, then plotted the signal counts in relation to the total number of β cell nuclei quantitated.

### mRNA-Sequencing and analysis

One hundred nanograms of RNA from Control and *Snd1* KO cell lines with RNA integrity numbers ≥ 7.0 was used for library following cDNA preparation (Takara) with the Nextera XT DNA Library Prep Kit (Illumina, Inc.). Each library was quantified, and quality accessed by Qubit and Agilent Bioanalyzer with average size of the library insert ~ 300–400 basepairs. Libraries were denatured and neutralized before loading to the NovaSeq 6000 sequencer for 100b paired-end sequencing (Illumina, Inc.). Approximately 12–18 × 10^6^ reads per library were generated. A Phred quality score (Q score) was used to measure the quality of sequencing with more than 95% of the sequencing reads reached Q30 (99.9% base call accuracy). The generated FASTQ files were processed using the Genialis visual informatics platform (https://www.genialis.com). Raw and analyzed mRNA-Sequencing data sets have been deposited in GEO (accession number: GSE240993).

### Quantitative PCR

RNA extraction from Control and *Snd1* knock out MIN6 cells was carried out following the manufacturer’s instructions (Zymo Research, D7001). Subsequently, cDNA synthesis was performed according to the manufacturer’s guidelines (Applied Biosystems 4,368,814). Quantitative (q)PCR reactions were conducted using the gene primers listed in Table 3 on a QuantStudio™ 3 Real-Time PCR System (Applied Biosystems, A28567). To determine relative gene expression changes, the 2^−ΔΔCT^ method was employed with 18s used as the normalization reference.

**Table 3.** qPCR primers.

Gene	Forward Primer	Reverse Primer
Mouse qPCR primers		
*18s*	AGTCCCTGCCCTTTGTACACA	GATCCGAGGGCCTCACTAAAC
*Glp1r*	ACGGTGTCCCTCTCAGAGAC	ATCAAAGGTCCGGTTGCAGAA
*Pdgfra*	AGAGTTACACGTTTGAGCTGTC	GTCCCTCCACGGTACTCCT
*Rgs8*	GCAGGAACAAAGGCATGAGGA	TGCTTCTTCCGTGGAGAGTCT
*Rgs16*	CCATGCCTTCCTAAAGACGGA	GTACTCGTCAAAGATGTGGTGAG
*Sct*	AGACACTCAGACGGAATGTTCA	CTGGTCCTCTAAGGGCTTGGA
*Six2*	CACCTCCACAAGAATGAAAGCG	CTCCGCCTCGATGTAGTGC
*Pou3f2*	GCAGCGTCTAACCACTACAGC	GCGGTGATCCACTGGTGAG
*Sp8*	GCTACCTGTAATAAGATCGGCAG	GAGGAGCGTTTCCAAGGGTG
*Crp*	ATGGAGAAGCTACTCTGGTGC	ACACACAGTAAAGGTGTTCAGTG
Human qPCR primers		
*18S*	GCAGAATCCACGCCAGTACAAG	GCTTGTTGTCCAGACCATTGGC
*PDX1*	AAGTCTACCAAAGCTCACGCG	GTAGGCGCCGCCTGC
*SND1*	CAGAACCGGCTTTCAGAATGT	TAGTATGTGAACCGTTCCCCT
*INS*	AGAGGCCATCAAGCAGATCACTGT	AGGTGTTGGTTCACAAAGGCTG
*GLP1R*	GGTGCAGAAATGGCGAGAATA	CCGGTTGCAGAACAAGTCTGT
*MAFA*	TGAGCGGAGAACGGTGATTTCTAAGG	GGAACGGAGAACCACGTTCAACGTA
*RGS16*	ATCAGAGCTGGGCTGCGATA	CAGGTCGAACGACTCTCTCC
*SIX2*	CCTGCGAGCACCTTCACAA	CTCGATGTAGTGTGCCTTGAG
*SLC2A1*	GGACAGGCTCAAAGAGGTTATG	AGGAGGTGGGTGGAGTTAAT
*SLC2A2*	CTAGGCAGAGCTGCGAATAAA	CTAGTTGGGAGTCCTGTCAATTC
*MAFB*	ACCTTGGCTAAGGCGAGAGTAG	CTTCAGCCTGGAGAGAAGTTACTC

### Glucose-stimulated insulin secretion

INS-1 832/13 and EndoC-βH1 cells treated with siControl and siSnd1/siSND1 were seeded at a density of 2 × 10^6^ cells per well in a 6-well plate. After either 48 (INS-1 832/13) or 72 (EndoC-βH1) hours, cells were exposed to a baseline glucose solution containing 1 mM glucose for 1 hour. The solution was then removed, and the cells were treated with either 2.8 mM glucose (low) or 16.7 mM glucose (high) for 1 hour. Secretion media was collected, and acid/ethanol solution was added to the cells to collect content samples. Measurement of INS-1 832/13 and EndoC-βH1 cell secretion media were performed using human insulin ELISA since INS-1 832/13 cells contain a human insulin expression cassette^24^. The ELISA was conducted by the Translation Core at Indiana University School of Medicine. To ensure consistency, all secretion samples were normalized to insulin content and fold change calculated by dividing the percentage of secreted insulin at high glucose to the percentage of secreted insulin at low glucose for each experimental replicate.

### Immunostaining

LUXendin551 (Celtarys Research; CELT-111; 250 nM) was applied to MIN6 *Snd1* knockout cells for 60 minutes before fixation in 4% paraformaldehyde. Mouse monoclonal α-E-cadherin 1:500 (BD Bioscience catalog 610,181) antibodies were applied overnight at 4°C in PBS + 0.1% Triton + 1% BSA. Secondary Cy5 donkey α-mouse (LI-COR IRDye secondary antibodies; 1:2000) antibodies were applied in the same buffer for 2 hours at room temperature. Images were acquired on a Zeiss LSM 800 confocal laser scanning scope and processed using ImageJ software. GLP1R surface expression was quantified vs. total E-cadherin expression and normalized against control cell lines.

### cAMP assay

cAMP assays were performed in *Snd1* knockout and control MIN6 cells. Briefly, 7500 cells/well were seeded into a 384-well plate and cells were incubated with 50 nM Exendin-4 in the presence of 16.7 mM glucose at 37°C in a humidified incubator with 5% CO_2_ for 30 minutes. Cell lysis and measurement of cAMP were carried out with a HTRF (Cisbio) assay according to the manufacturer’s instructions using a Molecular Devices SpectraMax iD5 plate reader. All assays were performed in the presence of 500 μM IBMX to inhibit phosphodiesterase activity.

### WST-1 assay

MIN6 control and *Snd1* knockout cell lines were used to prepare the cell suspension at a concentration of 5 × 10^4^ cells/well in 100 µl culture medium. The suspensions were then seeded in 96-well plates (100 µl/well) and incubated at 37°C in a humidified incubator with 5% CO_2_ for required exposure time. Each day following, WST-1 reagent solution was added to each well and the plate is incubated at 37°C for 2 hours. After incubation, the absorbance is measured at 450 nm with a multiplate reader.

### Statistical analysis

To determine statistical significance, the two-tailed Student’s t-test and/or One-way ANOVA were employed. The data are presented as the mean ± SEM. A significance threshold of *p* < 0.05 was used to identify significant differences between groups. Each experiment was repeated a minimum of three times.

## Results

### PDX1 and SND1 interact in mouse and human β cells

**Figure 1.**
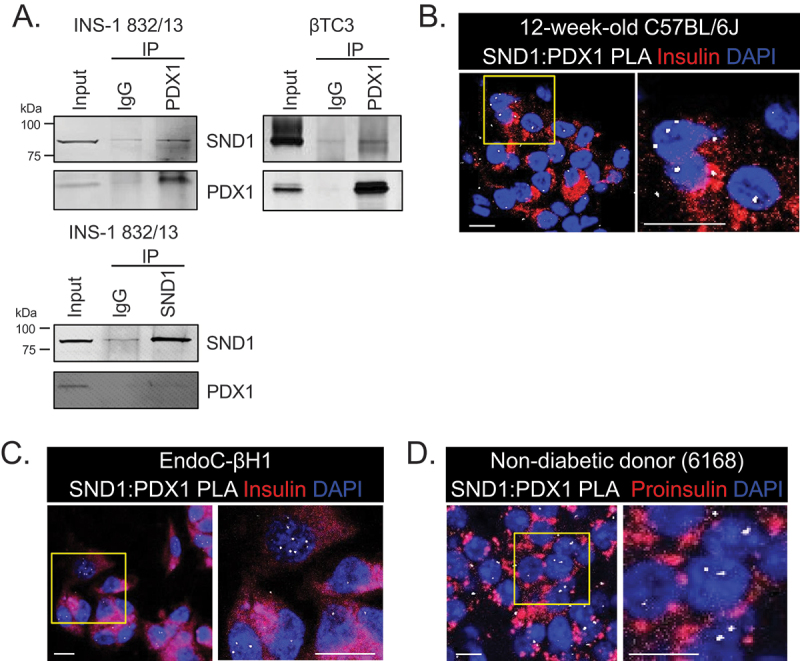
PDX1 and SND1 interact in rodent and human β cell lines and primary β cells. (a) co-immunoprecipitation of PDX1 or SND1, followed by immunoblotting for PDX1 and SND1 in INS-1 832/13 and βTC3 nuclear extracts. (b-d) proximity ligation assays (PLAs) using antibodies for SND1 and PDX1 were performed on (b) 12-week-old C57BL/6J, (c) human EndoC-βH1 β cells, and (d) 51-year-old non-diabetic male donor tissues acquired from nPOD (donor # 6168). The images on the right in b-d are magnified regions outlined in the yellow box. The white, fluorescent foci from the PLA represent individual PDX1:SND1 interactions. (scale bars = 10 μm).

To validate the novel PDX1:SND1 interactions discovered through unbiased IP-mass spectrometry analysis using nuclear enriched extract from rodent pancreatic β cell lines (Personal Communication, Roland Stein, Vanderbilt University (April 2022)), we conducted co-immunoprecipitation (co-IP) experiments on non-crosslinked nuclear enriched extracts from two different rodent lines: βTC3 and INS-1 832/13 cells. For the co-IP, we employed PDX1 and SND1 antibodies to capture the complexes associated with the proteins and then immunoblotted for SND1 and PDX1. The results revealed that SND1 and PDX1 proteins are pulled-down in the reciprocal co-IP labels in comparison to the IgG controls, thereby confirming the interaction between these proteins (Figure 1a).

To further validate the presence of PDX1:SND1 complex formation in β cell nuclei, we conducted proximity ligation assays (PLA) using α-SND1 and α-PDX1-specific antibodies in 12-week-old C57BL/6J mouse pancreatic tissue (Figure 1b), human EndoC-βH1 β cell lines (Figure 1c), and pancreatic tissue from a non-diabetic human donor acquired from the Network for Pancreatic Organ Donors (nPOD) (Figure 1d; nPOD donor: 6168). The PLA assay generates fluorescent puncta if the target proteins are within 30–40 nm of each other, indicating protein-protein interactions. As a control for the PLA, we performed PLA using α-PDX1-specific antibody alone demonstrating minimal background signals (Supplemental Figure S1). In both mouse and human β cell nuclei incubated with both PDX1 and SND1 antibodies we observed PLA signals, providing further confirmation of the presence of PDX1:SND1 complex formation in primary β cells.

### Glucose-stimulated insulin secretion is impaired in SND1-depleted rodent and human β cells

**Figure 2.**
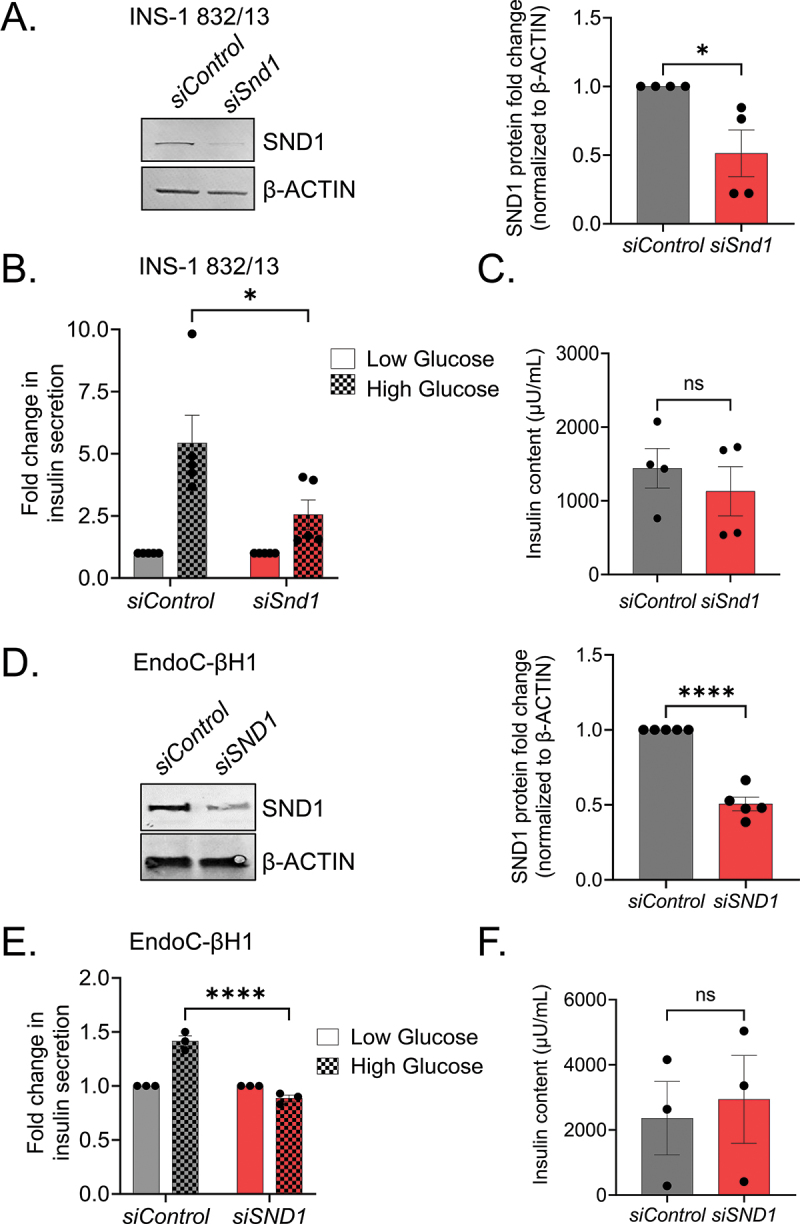
Reduction in *Snd1* negatively impacts glucose-stimulated insulin secretion in rodent and human β cell lines. Immunoblot of nuclear extracts from siControl and si*Snd1* treated INS-1 832/13 (a) and EndoC-βH1 (d) cells demonstrate significant reductions of SND1. Quantitation of SND1 protein levels normalized to β-ACTIN shown to the right. (b) glucose-stimulated insulin secretion in si*Snd1* cells is blunted in INS-1 832/13 (b) and EndoC-βH1 (e) cells with no change in insulin content (c, f). (*n* = 3–5). ns, not significant; *, *p* < 0.05; ****, *p* < 0.0001.

Pancreatic β cells are unique in synthesizing, packaging and releasing insulin upon glucose stimulation. To determine if SND1 depletion affects glucose stimulated insulin secretion (GSIS) in β cells, we knocked down *Snd1* via siRNA in the rat INS-1 832/13 and human EndoC-βH1 β cell lines, which achieved 51 ± 31% (INS-1 832/13) or 50 ± 8.2% (EndoC-βH1) knockdown (Figures 2a, 2d respectively). In response to 16.7 mM high glucose challenge, secreted insulin levels in the siSnd1/siSND1 knockdown cells were significantly lower than siControl cells Figure 2(d,e). Insulin content in *siSnd1* knockdown cell lines remained unchanged suggesting SND1 is not critical for insulin production Figure 2(c-f).

### SND1 regulates important genes related with insulin secretion in rodent β cell lines

**Figure 3.**
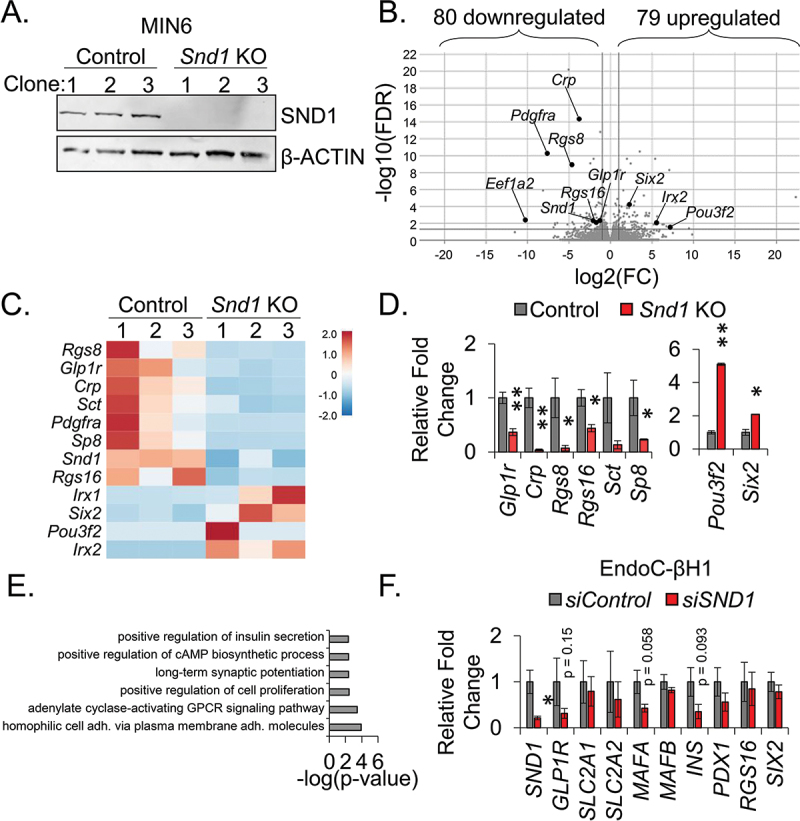
SND1 controls a subset of genes important for β cell expansion and function. (a) immunoblot of nuclear extracts for SND1 and β-ACTIN from clonal CRISPR/Cas9 *intron* - control and *exon 2* - *Snd1* KO cell lines. (b) volcano plot illustrating the most differentially expressed genes in *Snd1* KO β cell lines. (c Heatmap hierarchical clustering displaying log2(Fold change) of select subset of differentially expressed genes in *Snd1* KO β cells. (d) qPCR performed on independently isolated RNA from control and *Snd1* KO cell lines. (e) biological processes of the 159 differentially expressed genes identified by gene ontology (GO) analysis include those associated with insulin secretion, cAMP biosynthesis, and cell proliferation. (f) qPCR of select subset of genes following siRNA-mediated depletion of SND1 from EndoC-βH1 cells. *, *p* < 0.05; **, *p* < 0.01.

To generate tools to investigate the defect in GSIS observed in rodent and human β cell lines following reduction of *Snd1*, we utilized CRISPR/Cas9 gene-editing technology to delete *Snd1* from MIN6 β cell lines. Previously generated Cas9 expressing MIN6 β cell lines^23^ were infected with lentivirus encoding gRNAs targeting *exon 2* coding region or a non-coding *intron* region (used as control) of *Snd1* (Table 1). Following puromycin selection, individual cells were seeded and expanded to derive clonal cell lines deficient for *Snd1*. Of the clonal lines screened, we selected 3 intron-targeted control and 3 *exon* 2-target SND1-deficient clonal cell lines (hereafter, referred to as *Snd1* KO) (Figure 3a). Sequencing of *Snd1* KO clones confirmed indels that led to disrupted SND1 protein (Supplemental Figure S2).

To identify genes differentially expressed in *Snd1* KO β cell lines, we performed unbiased mRNA sequencing and based differential gene expression on a two-fold cutoff and false discovery rate of < 0.05. With these parameters, we discovered 80 downregulated- and 79 upregulated-genes Figure 3(b,c). qPCR analysis was performed to validate several of the differentially downregulated (*Glp1r, Crp, Rgs8, Rgs16, Sct*, and *Sp8*) and upregulated genes (*Pou3f2, Six2*) (Figure 3d). Gene Ontology (GO) analysis of these genes using the Database for Annotation, Visualization, and Integrated Discovery (DAVID) led to the identification of pathways linked to cell proliferation, cAMP biosynthesis and insulin secretion (Figure 3e), the latter of which could explain the functional defect in insulin secretion following the *Snd1* knockdown in INS-1 832/13 and human EndoC-βH1 β cell lines (Figure 2). Interestingly, qPCR analysis in EndoC-βH1 cells following *SND1* knockdown revealed a partial overlap with genes differentially expressed in *Snd1* KO cells, including a trending reduction in *GLP1R*, and unique differences between mouse and human cell lines, including trending reductions in *MAFA* and *INS* (Figure 3f). We next utilized publicly available PDX1-ChIPSeq datasets from mouse islets peaks localized to 7876 genes^25^ and overlaid with the 159 differentially expressed genes identified in *Snd1* KO cells. This analysis revealed 59 of the 159 DEGs in *Snd1* KO cells were bound by PDX1 in mouse islets including *Glp1r, Crp, Rgs8, Rgs16.*

Interestingly, *SND1* has been linked to cell proliferation in the context of human cancer progression and metastatic spread^26^. We followed up on our GO pathway analyses findings by performing a WST-1 assay for cell expansion. Control and *Snd1* KO cells were seeded at the same density and the WST-1 reagent was added on sequential days to measure expansion. The cleavage of the tetrazolium salt WST-1 to formazan by cellular mitochondrial dehydrogenases is measured as a proxy for the number of viable cells in the dish. Interestingly, we revealed that *Snd1* KO lines have significantly blunted cell expansion capacity in comparison to control lines (Supplementary Figure S3A).

### SND1 deficient rodent β cell lines show a trend towards reduced GLP1R and cAMP response to exendin-4

GLP1R is a class B G-protein-coupled receptor involved in glucose homeostasis and serves as a recent modality for treating T2D and obesity^27^. GLP-1, an incretin that targets GLP1R, has been shown to have several effects, including potentiating insulin secretion, inhibiting glucagon secretion, slowing gastric emptying, and promoting satiety^28^. Reagents for the detection of GLP1R protein in tissues are limited. Recently, GLP1R fluorescent antagonists (termed LUXendins) have been generated to efficiently label GLP1R on the surface of tissues in which it is expressed^29,30^. To evaluate changes in GLP1R protein levels, *Snd1* KO and control β cell lines were treated with the fluorescent LUXendin 551 probe and counterstained with E-cadherin to mark the surface of the β cells. Quantitative analyses revealed that GLP1R levels are trending down in *Snd1* KO cell lines (Supplemental Figure S3B).

Upon agonist binding to the GLP1R on the surface of pancreatic β cells, downstream signaling leads to activation of the enzyme adenylate cyclase, which converts ATP into cyclic adenosine monophosphate (cAMP)^31,32^. Increased cAMP levels play a crucial role in the potentiation of GSIS by enhancing insulin granule exocytosis and gene expression of insulin and other factors involved in insulin secretion^33^. Given the trending reduction of GLP1R protein in *Snd1* KO cells (Supplemental Figure S3B) and the link of other genes differentially expressed to cAMP biosynthesis (Figure 3E), we measured cAMP accumulation in control and *Snd1* KO β cell lines upon high glucose (16.7 mM) in presence of GLP1R-agonist, Exendin-4. We observed a trending reduction in cAMP accumulation in the *Snd1* KO cell lines as compared to control cell lines (Supplemental Figure S3C).

### PDX1:SND1 interactions are reduced in T2D donor β cells

**Figure 4.**
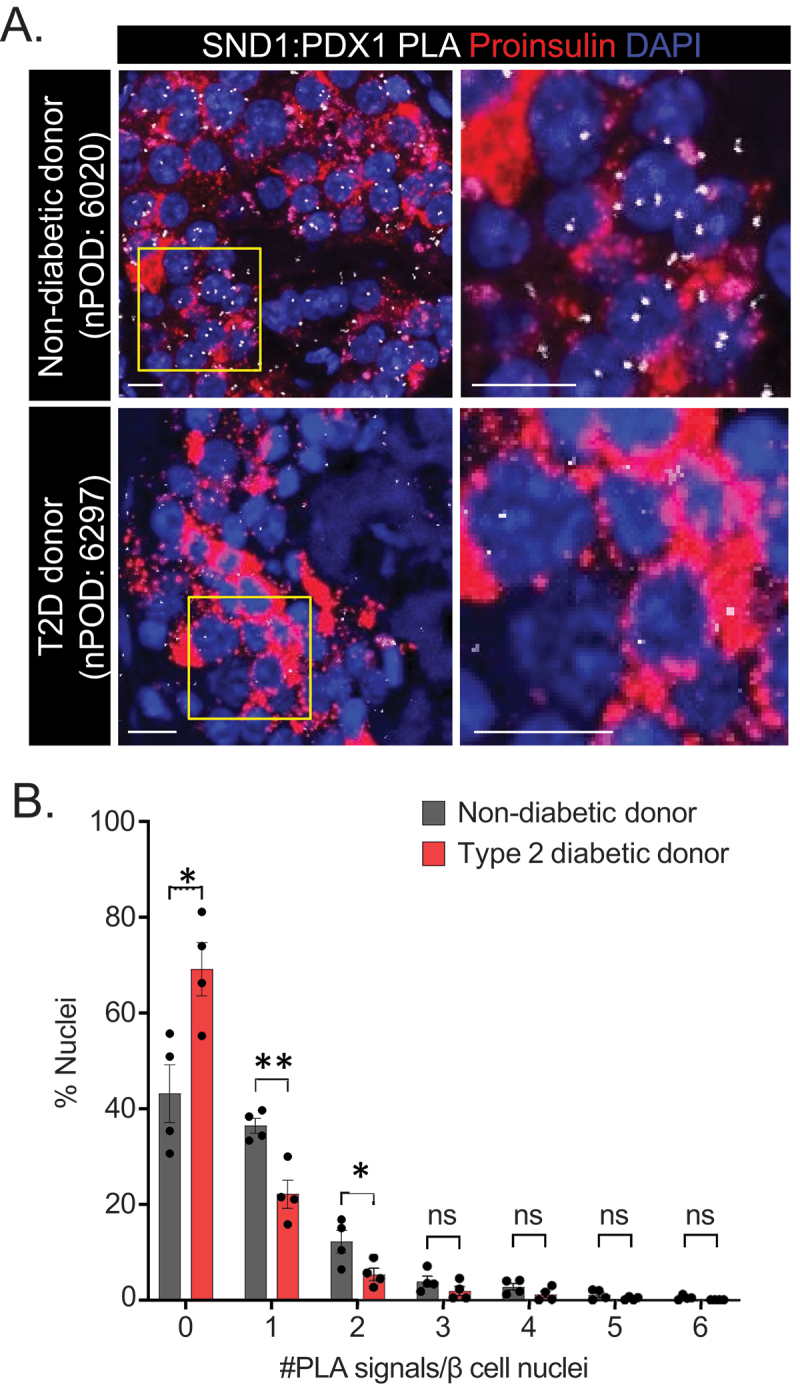
PDX1:SND1 interactions are negatively impacted in T2D human β cells. (a) Representative PDX1:SND1 PLA and proinsulin images acquired from human pancreatic tissue sections from non-diabetic donor (nPOD case #: 6020: 60 years old male, BMI = 29.8) and T2D donor (nPOD case#: 6297: 60 years old male, BMI = 29.5, 3 years T2D duration). (b) quantitation of PLA signals in each group stratified by number of PLA signals per β cell nuclei. The images to the right in (a) are magnified regions outlined in the yellow box above. (*n* = 4). ns, not significant; *, *p* < 0.05; **, *p* < 0.01. (scale bars = 10 μm).

Previously, our group and others have revealed that interactions between PDX1 and the coregulators BRG1 and CHD4 are reduced in human T2D donor β cells^13,23^, indicating an important role for the PDX1:BRG1 and PDX1:CHD4 complex formation to maintain β cell function. To assess whether PDX1:SND1 demonstrate similar perturbed interactions, we utilized PLA on closely age-, BMI- and sex-matched non-diabetic and T2D human donor tissue sections procured from nPOD (Donor information in Table 2, *n* = 4). We observed that a greater number of β cell nuclei were devoid of PLA signals and fewer β cell nuclei contained 1 and 2 PLA signals in T2D donor tissue compared to non-diabetic donor tissue Figure 4(a,b). We confirmed that PDX1 and SND1 protein levels were similar between non-diabetic and diabetic donor tissues, inferring the reduced interaction is not due to less protein in the samples (Supplemental Figure S4). These data highlight the importance of PDX1:SND1 interactions in maintaining β cell function, and that disruption of their interactions is associated with β cell dysfunction present in T2D pathophysiology.

## Discussion

Gene regulation by PDX1 (and other TFs) is principally coordinated through protein-protein interactions between DNA-bound TFs and recruited coregulators, which positively or negatively impact transcription. There is a glaring need to understand how PDX1 transcriptional activity is controlled by coregulators under physiological conditions in the β cell, as this will lead to a deeper understanding of its failure in T2D. In this regard, PDX1 was shown previously, and recently by our group, to interact with a diverse set of transcriptional coregulators in the β cell, several of which have been shown to modulate its activity. For example, the ATP-dependent SWI/SNF chromatin remodeling complex positively contributes to PDX1 by regulating gene expression of a subset of PDX1 target genes critical for β cell function and identity *in vivo*^14^. We have also shown the importance of PDX1:CHD4 interactions in maintaining pancreatic β cell function in both *in vitro* ^*23*^ and *in vivo* ^*15*^ studies. Here we have defined an important role for SND1, a newly defined PDX1-interacting partner, in controlling a subset of genes critical for β cell function.

SND1 has been implicated in a variety of cellular functions including RNA metabolism^34^, scaffolding for protein-protein interactions^21,35^, nuclease functions, miRNA processing^36^, and transcription regulation^19,20,37^. Accordingly, with its diverse functional roles, SND1 protein is detected in multiple intracellular locations, including the endoplasmic reticulum^38^, stress granules^39^, lipid droplets^40^, and nuclei^41^. We have also observed this using the tools at our disposal, with SND1 being present in nuclear enriched extracts of β cell lines (Figures 1–3), immunofluorescence staining demonstrating a cytoplasmic and nuclear presence of SND1 (Supplemental Figure S4), and PLA puncta with PDX1:SND1 present throughout β cell nuclei (Figures 1,4).

Given the role of SND1 in pivotal biological processes like regulation of gene expression, cell cycle and overexpression in cancer setting (breast cancer, colon cancer, prostate cancer, lung cancer and liver cancer)^22,42–45^, we suspected SND1 would play an important role in regulating genes that are linked to β cell expansion. Our RNA-sequencing data support this idea as GO pathway analysis of the differentially expressed genes in *Snd1* KO cells are linked to cell proliferation (Figure 3e). Moreover, we confirmed that *Snd1* KO cells have limited cell expansion properties in comparison to control cell lines (Supplemental Figure S3A). We predict these defects are likely due to proliferation delays, since should SND1 be involved in restraining apoptosis in the β cell, *Snd1* KO cell lines would have never thrived during expansion of initial single cell seeding to generate the lines. Our current study is limited to utilizing *in vitro* cell line model systems where *Snd1* is transiently reduced using siRNAs or knocked out by CRISPR technology. Future efforts on the role of SND1 in β cell expansion will be addressed using *in vivo* model systems.

One of the most profound functional defects observed was the limited ability of *Snd1* knockdown cell lines to secrete insulin under high glucose stimulatory conditions. We speculate this is likely a result of glucose processing and/or insulin exocytosis defects, as we did not observe changes in INSULIN processing enzymes in RNASeq datasets and INSULIN content is unchanged (Figures 2c, 2f, 3). Gene expression and GO pathway analyses of *Snd1* KO cell lines support a transcription mediated defect that leads to these changes, including reduced expression of *Glp1r, Rgs8* and *Rgs16*, the latter of which has been directly implicated as a positive regulator of insulin secretion and β cell proliferation^46,47^. Moreover, we found that both GLP1R protein and cAMP production following exendin-4 stimulation was trending down in *Snd1* KO β cell lines (Supplemental Figure S3B), further linking the gene expression changes following *Snd1* deficiency to those functional aspects of β cell biology. The potential role of *Snd1* in this pathway is especially relevant as exendin-4 has been utilized to improve glycemic outcomes in individuals with T2D. Interestingly, we observed some gene expression differences between *Snd1* KO cell lines and *siSND1* EndoC-βH1 cells. For example, we observed trending reductions in *INS* and *MAFA* genes in *siSND1* EndoC-βH1 cells; however, these transcripts were unchanged in *Snd1* KO cells and INSULIN content was unaltered in both cell lines (Figures 2c, 2f, 3).

T2D is a complex, polygenic disease with a heterogenous pathophysiology, associated with dysfunctional β cells^48^ and reduced activity of important TFs^12^. In this regard, our lab and others have previously established that interactions between PDX1 and its associated coregulators BRG1^13^ and CHD4^23^ are perturbed in T2D β cells, findings which highlight the underappreciated role of coregulators in pathogenesis of T2D. Here we extend upon the impact of coregulators on T2D pathogenesis and demonstrate loss of interactions between PDX1 and SND1 in T2D human donor β cells (Figure 4). As with most studies using T2D donor tissues, we are limited to inquiring about these interactions after the onset of the disease. It will be interesting to combine such investigations and compare the results of the dynamic changes related to PDX1:SND1 interactions overtime in a T2D rodent model system during the onset and progression of the disease. Past studies^13,23^ and our report here, uncover that PDX1 loses its ability to interact with important coregulators in T2D β cells. As PDX1 is the common factor in these analyses, it suggests that PDX1 itself undergoes a detrimental change that limits ability to recruit coregulators during the development of T2D. Future efforts on what modifications occur to PDX1 (i.e., posttranslational modifications) during T2D pathogenesis, prior to significant reductions in PDX1 protein levels, would be of significant value to understanding the mechanisms of β cell failure in T2D.

Overall, our results demonstrate a critical role of SND1 in controlling insulin secretion and modulating a subset of genes important for β cell function. We have discovered that PDX1 interacts with SND1 in the nuclei of pancreatic β cells both *in vitro* and *in vivo* and these interactions are negatively impacted in pathophysiological settings of T2D. These findings are the first to demonstrate that SND1 plays an essential role in maintaining β cell function and provide evidence that loss of PDX1:SND1 complex formation is a significant feature of diabetes pathophysiology. Future studies will be focused on dissecting the molecular mechanisms by which SND1 is able to regulate insulin secretion and proliferation in β cells using *in vivo* model systems.

## Supplementary Material

Supplemental Material
