# Supplementary material for: Dynamic regulation of pancreatic β cell function and gene expression by the SND1 coregulator in vitro
Source: Islets. 2023 Oct 15;15(1):2267725. doi: 10.1080/19382014.2023.2267725 (PMC10578191; doi:10.1080/19382014.2023.2267725)

Supplemental Figure 1: PDX1 antibody alone produces minimal PLA signals in human β cells.

EndoC-βH1

PDX1 PLA Insulin DAPI

Non-diabetic donor

(nPOD: 6254)

PDX1 PLA Insulin DAPI


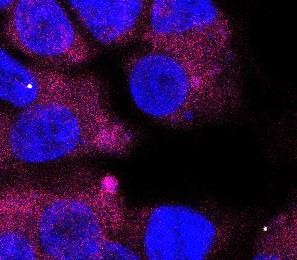

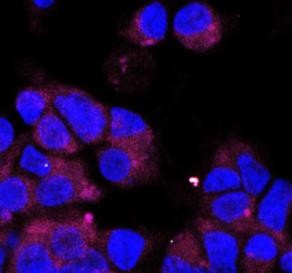

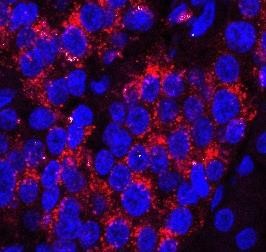

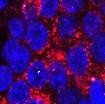


Supplemental Figure 2: CRISPR/Cas9 mediated gene editing of *Snd1* leads to indels that disrupt open reading frame of *Snd1* Exon 2.

|  | **Exon 2 Sequence** | | | | | | | | | | | | | | | | | | | | | | | | | | | | | | | | | | | | | | | | | | | | | | | | | | | | | | | | | | | | | |  |
| --- | --- | --- | --- | --- | --- | --- | --- | --- | --- | --- | --- | --- | --- | --- | --- | --- | --- | --- | --- | --- | --- | --- | --- | --- | --- | --- | --- | --- | --- | --- | --- | --- | --- | --- | --- | --- | --- | --- | --- | --- | --- | --- | --- | --- | --- | --- | --- | --- | --- | --- | --- | --- | --- | --- | --- | --- | --- | --- | --- | --- | --- | --- | --- |
| **WT** | **V** | | | **L** | | | **S** | | | **G** | | | | **C** | | | **A** | | | | **I** | | | **I** | | | **V** | | | **R** | | | **G** | | | **Q** | | | **P** | | | **R** | | | **G** | | | **G** | | | **P** | | | **P** | | | **P** | | | **E** | | | **…** |
| **Snd1 KO1** | **V** | | | **L** | | | **S** | | | **G** | | | | **C** | | | **A** | | | | **S** | | | **L** | | | **A** | | | **G** | | | **P** | | | **P** | | | **P** | | | **P** | | | **T** | | | **E** | | | **R** | | | **P** | | | **S** | | | **T** | | | **…** |
| **Snd1 KO2** | **MAJOR DELETION** | | | | | | | | | | | | | | | | | | | | | | | | | | | | | | | | | | | | | | | | | | | | | | | **G** | | | **E** | | | **I** | | | **S** | | | **L** | | | **…** |
| **Snd1 KO3** | **V** | | | **L** | | | **S** | | | **G** | | | | **C** | | | **A** | | | | **I** | | | **I** | | | **V** | | | **R** | | | **G** | | | **Q** | | | **S** | | | **P** | | | **R** | | | **M** | | | **I** | | | **I** | | | **L** | | | **V** | | | **…** |
|  | | | | | | | | | | | | | | | | | | | | | | | | | | | | | | | | | | | | | | | | | | | | | | | | | | | | | | | | | | | | | | | |
|  |  | | | | | | | | | | | **gRNA1** | | | | | | | | | | | | | | | | | | | | | **PAM** | | |  | | | | | | | | | | | | | | | | | | | | | | | | | | | |
| WT Exon 2 | G | T | C | C | T | C | T | C | T | G | | G | G | T | G | C | G | | C | C | A | T | A | A | T | T | G | T | C | C | G | A | G | G | G | C | A | G | C | C | C | C | G | G | G | G | T | G | G | T | C | C | T | C | C | T | C | C | T | G | A | G | **…** |
| Snd1 KO1 | G | T | C | C | T | C | T | C | T | G | | G | G | T | G | C | G | | C | C | T | C | C | C | T | G | G | C | C | G | G | G | C | C | T | C | C | T | C | C | T | C | C | T | A | C | G | G | A | G | A | G | G | C | C | C | T | C | A | A | C | C | **…** |
| Snd1 KO2 | MAJOR DELETION | | | | | | | | | | | | | | | | | | | | | | | | | | | | | | | | | | | | | | | | | | | | | | | G | G | G | G | A | G | A | T | C | T | C | T | C | T | G | **…** |
|  | | | | | | | | | | | | | | | | | | | | | | | | | | | | | | | | | | | | | | | | | | | | | | | | | | | | | | | | | | | | | | | |
|  |  | | | | | | | | | | | | | | | | | | | | | | **gRNA2** | | | | | | | | | | | | | | | | | | | | **PAM** | | |  | | | | | | | | | | | | | | | | | |
| WT Exon 2 | G | T | C | C | T | C | T | C | T | | G | G | G | T | G | C | G | C | | C | A | T | A | A | T | T | G | T | C | C | G | A | G | G | G | C | A | G | C | C | C | C | G | G | G | G | T | G | G | T | C | C | T | C | C | T | C | C | T | G | A | G | **…** |
| Snd1 KO3 | G | T | C | C | T | C | T | C | T | | G | G | G | T | G | C | G | C | | C | A | T | A | A | T | T | G | T | C | C | G | A | G | G | G | C | A | A | T | C | C | C | C | C | C | G | G | A | T | G | A | T | C | A | T | C | C | T | C | G | T | G | **…** |

Supplemental Figure 3: Snd1 deficiency reduces cell expansion, GLP1R and cAMP levels


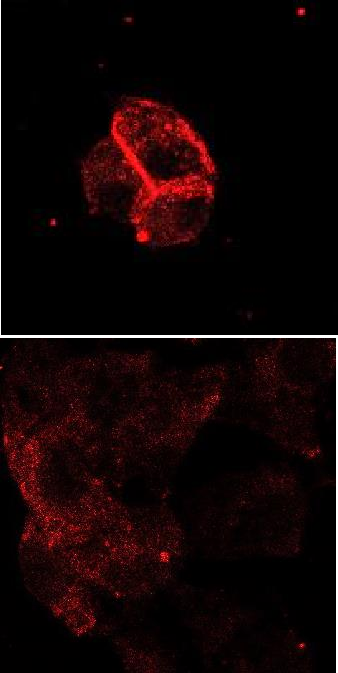

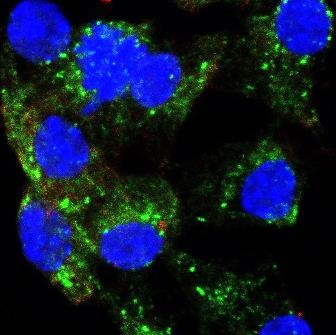

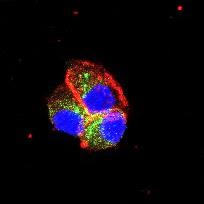


LUXendin551

LUXendin551 E-cadherin DAPI

6


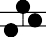

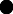

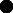

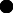


0.0719

Corrected Total Fluorescence Intensity Ratio (CTFR)

4

Control

2

0

*Snd1* KO

Control *Snd1* KO

1. cAMP accumulation

0.2384

50 nM Ex-4-stimulated cAMP (Fold 16.7G condition)

20


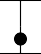


15

10

5

0

Control *Snd1* KO

Supplemental Figure 4: SND1 and PDX1 levels are unchanged in non-diabetic

PDX1

SND1

and T2D human donor tissues.


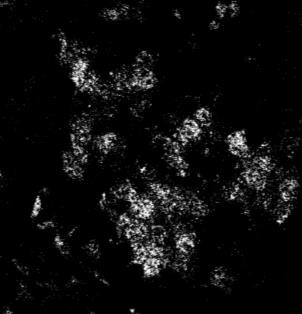

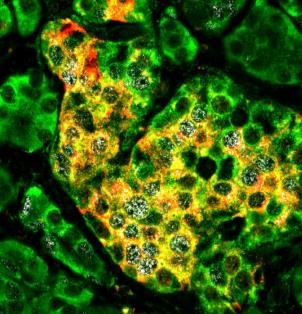

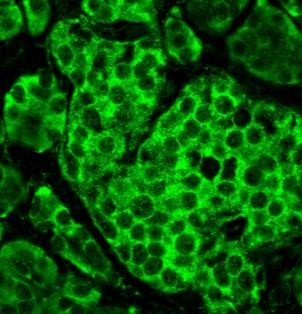


PDX1 SND1

Proinsulin

Non-diabetic (nPOD: 6020)

T2D (nPOD: 6255 )


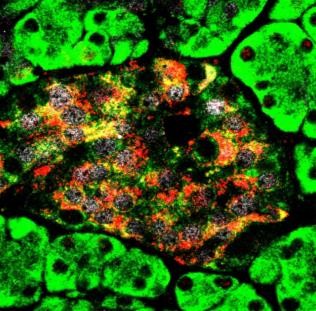

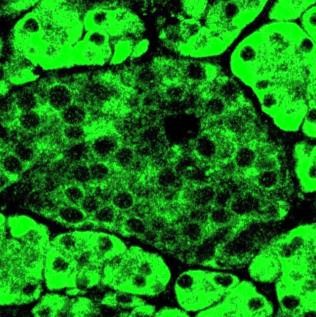

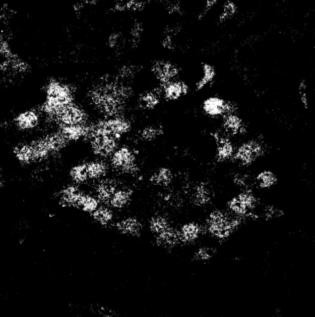

Supplement: Supplemental Material [file KISL_A_2267725_SM3998.docx]
